# A Case Report of Excessive Use of Clozapine Combined With Clonazepam

**DOI:** 10.3389/fpsyt.2022.831276

**Published:** 2022-02-15

**Authors:** Wei Li, Yan Liu, Haifeng Jiang, Jiang Du, Yan Zhao, Zheyi Du, Shuo Li, Haihong Wang

**Affiliations:** ^1^Shanghai Mental Health Center, School of Medicine, Shanghai Jiaotong University, Shanghai, China; ^2^Alzheimer's Disease and Related Disease Center, Shanghai Jiaotong University, Shanghai, China; ^3^Shanghai Baoshan Mental Health Center, Shanghai, China; ^4^Shanghai Mental Health Center Clinical Research Center, Shanghai, China

**Keywords:** schizophrenia, clozapine, clonazepam, therapeutic drug monitoring (TDM), withdrawal, case report

## Abstract

**Introduction:**

For patients with schizophrenia, clozapine (CLZ) in combination with clonazepam (CLNAZ) is one of the viable therapeutic options. We successfully reduced the doses of CLZ and CLNAZ to the safe range of a polydrug abuse patient. As far as we know, this is the first case of this problem. As there are no relevant guidelines to reduce CLZ or CLNAZ, we hope to share this case to provide a reference for the prevention and treatment of similar patients with multidrug abuse.

**Case Presentation:**

This case report describes a 46-year-old male with a 24-year history of schizophrenia. His main clinical manifestations are auditory hallucinations, persecutory delusion, and emotional instability. In 2012, the patient started taking rifampicin due to tuberculosis and gradually overused CLZ and CLNAZ. Before admission, he took 1,275 mg of CLZ every day and 26 mg of CLNAZ every night. With the help of Therapeutic Drug Monitoring (TDM) and pharmacogenetic testing, we gradually reduced his daily dose of CLZ and CLNAZ and formulated a more reasonable dosing schedule for him. At the time of discharge, the patient took CLZ 450 mg per day and CLNAZ 2 mg per night, with no obvious symptoms of psychosis.

**Conclusion:**

In the process of drug maintenance treatment of schizophrenia, it is necessary to adopt TDM strategy to reduce and treat the abuse of multiple prescription drugs.

## Introduction

The problem of misuse/abuse/dependence of prescribed medications (e.g., prescription stimulants, sedatives and hypnotics, antipsychotics, etc.) is a highly topical and interesting issue (1). Clozapine (CLZ) is a widely used antipsychotics in China, and it is also a recommended first-line medication for refractory schizophrenia. However, contrary to the wide use of CLZ, the side effects of CLZ include agranulocytosis, seizures, antipsychotic malignant syndrome, salivation, arrhythmia, changes in consciousness, myocarditis, diabetic ketoacidosis, gastrointestinal motility, which can belife-threatening (2). Although not common, there have been reports of abuse of CLZ, which may be attributed to the effectiveness of the drug (3). Overdose of CLZ may cause drowsiness, agitation, irritability, hallucinations, confusion, slurred speech, and even death (4). However, once CLZ is discontinued improperly or suddenly, adverse reactions may occur, including but not limited to discontinuation-related psychosis, cholinergic rebound, catatonia, serotonin discontinuation symptoms, abnormal blood quality, and suspected myocarditis (5).

CLZ is sometimes used in combination with CLNAZ in patients with schizophrenia. Clonazepam (CLNAZ), as a benzodiazepine sedative and broad-spectrum antiepileptic, can be used to treat sleep disorders, reduce anxiety and improve sleep quality (6). Past studies have shown that this combination may affect the blood concentration of Norclozapine (N-CLZ), there by affecting the therapeutic effect (7). Potential problems with CLNAZ abuse include physical and psychological dependence, suicidal thoughts or behavior, worsening depression, sleep disturbance, and aggression.

Despite the severe effect of overdosing CLZ and CLNAZ, there are no guidelines to adjust the amount of CLZ or CLNAZ in patients who abuse both medications. Worse still, random dose change can cause severe and even life-threatening withdrawal syndromes. Although we can guide our medication by monitoring the blood concentration of CLZ, many physiological factors or drug combinations will affect the blood concentration of CLZ, such as age, gender, smoking (8), fluvoxamine and ciprofloxacin Star (9). Therefore, the withdrawal of the drugs has brought massive challenge to clinical treatment, which is worthy of being studied.

This article describes a case of combined abuse of CLZ and CLNAZ in a patient with schizophrenia, and the process of adjusting its dosing regimen using TDM strategies.

## Case Presentation

The patient, male, 46 years old, married, college degree, bank clerk, has a history of schizophrenia for 24 years. In 1997, he was admitted to a mental hospital due to suspiciousness, hallucinations, delusions of persecution, emotional instability, insomnia, and behavioral disorders. After that, he was treated with CLZ (150 mg per day) and his symptoms were well controlled. In 2009, due to the discontinuation of CLZ treatment, he relapsed and was hospitalized again. This time, he showed basically the same symptoms as last admission. The dose of CLZ gradually increased to 6–14 tablets (25 mg per tablet, 500 mg/day). His psychiatric symptoms were well controlled, but his sleep was still poor, so he started taking alprazolam, gradually increasing the dose from 1 tablet to 5 tablets (0.4 mg per tablet, 2 mg/night). Later, his sleep improved very well, and the patient was discharged after getting better. After being discharged from the hospital, the patient took the medication on time.

By 2012, the patient had insomnia again. To enhance sleep quality, he changed alprazolam to CLNAZ as prescribed by the doctor and gradually increased from 1 tablet to 4 tablets (each 2 mg, 8 mg/night). The patient then received rifampicin for treatment of tuberculosis. The patient felt that the drugs were not as effective as before, so he increased CLZ to 6–26 tablets (25, 800 mg/day), and CLNAZ from 4 tablets to 13 tablets (2, 26 mg/night). He has been taking the above dose for 3 years.

In April 2021, the patient recovered from tuberculosis and stopped rifampicin, but still had poor sleep quality, and again increased the doses of CLZ and CLNAZ by himself. About 2 months before admission, he took CLZ 6–45 tablets (25 mg per tablet, 1,275 mg per day) and 13 CLNAZ tablets (2 mg per tablet, 26 mg per night). He did not feel any particular discomfort. In order to adjust the dosage of the drug, he was admitted to our hospital for treatment on May 26, 2021. The patient signed an informed consent upon admission. Due to routine medical care, the approval of the ethics committee is not required.

He reported no history of heart or respiratory disease, diabetes, high blood pressure, epilepsy, or drug allergy. The patient did not had a history of druge abuse/misues or previous excessive use of benzodiazepine.

## Investigation

**Figure 1:**
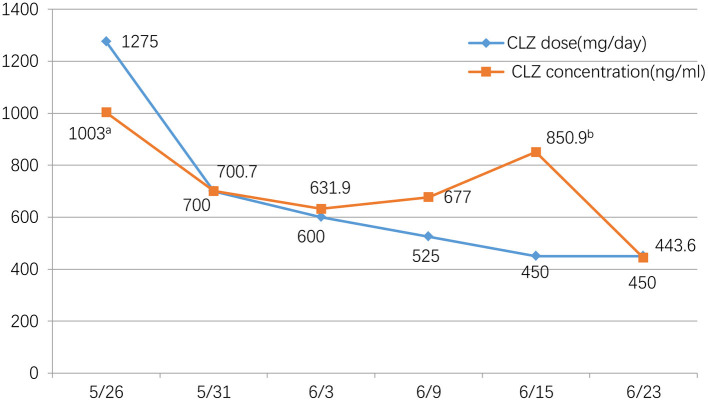
Clozapine dose(mg/day) and concentration (ng/ml) during the tapering period. We monitored the CLZ concentration approximately twice a week. We chose a fixed time, every morning at 6:30, before breakfast, to take blood samples. ^a^At the time of the initial evaluation, when the patient was admitted at 13:00, 17 h after the last medication. ^b^For some reasons, the patient did not draw blood at 6:30 in the morning, but at 10:30, so this value is discrepant from others.

At the time of the initial evaluation, 17 h after the last medication, the patient was conscious but had mild dysarthria, with withdrawal symptoms such as mild tremor, sweating, and palpitations. Vital signs: blood pressure 120/80, pulse 112, respiration 18, body temperature 36.4°C. Physical examination revealed involuntary hand tremor, and other physical examination results were normal. Blood counts, liver function, kidney function, serum electrolytes, serum prolactin, hormones, thyroid function tests, urinalysis and Head Magnetic Resonance Imaging (MRI) were normal. Creatine kinase 1135U/L↑. Electroencephalogram (EEG) showed θ frequency-domain power increased, mild abnormalities. Electrocardiogram (ECG) showed sinus tachycardia, heart rate 112 beats per minute. Color ultrasound of the abdomen showed severe distension of the abdomen. Computerized Tomography (CT) scan of the chest showed scattered inflammation in the posterior segment of the right upper lobe. The serum CLZ concentration was determined by high-pressure liquid chromatography (Table 1; Figure 1). N-CLZ and CLNAZ were not detected in the hospital. Pharmacogenetics test: CYP1A2 ultra-rapid metabolizer, CYP2C19 extensive metabolizer, CYP3A4 extensive metabolizer. Several scales were evaluated, including Self-Rating Depression Scale (SDS) (score 42, mild depressive symptoms), Self-Rating Anxiety Scale (SAS) (score 30, no obvious anxiety symptoms), EPQ personality test (moderate introverted personality, typical stable Emotional stability), Clinical Institute Withdrawal Assessment scale-Benzodiazepines (CIWA-B) (9 points, mild withdrawal) (Table 1), Negative Syndrome Scale (PANSS) (50 points, showing low mood, paying attention to physical health, self-awareness) (Table 1).

**Table 1:** Changes in dosage of clozapine (CLZ), clonazepam (CLNAZ) and trazodone, the rating scale score of Clinical Institute Withdrawal Assessment scale-Benzodiazepines (CIWA-B), Positive And Negative Syndrome Scale (PANSS), and the serum concentration of CLZ (ng/ml).

**Date**		**5/25**	**5/26**	**5/28**	**5/30^**#**^**	**5/31**	**6/3**	**6/6**	**6/9**	**6/12**	**6/15**	**6/22^**&**^**	**6/23**	**7/1**
CLZ (tablets)		6–45	4–30	4–27	4–27	4–24	4–20	4–17	4–14	4–12	4–14	4–14	6–12	6–12
PANSS score		/	50	/	/	/	51	/	/	56	/	/	51	50
CLNAZ (tablets)		13	10	9	9	8	6	5	4	3	2	2	1	1
CIWA-B		/	9	/	10	/	12	/	/	18	/	/	16	11
Trazodone (mg)		/	/	/	/	/	/	/	/	/	/	/	50	50
Clozapine	6:30*					700.7	631.9		677.0		850.9^b^		443.6	
concentration	13:00		1,003.0^a^											
(ng/ml)*	19:59 20:30 21:00 22:00 23:00				377.0 373.0 1,189.4 1,433.2 /							187.3 / 739.5 702.6 574.8		

*We monitored the CLZ concentration approximately twice a week. We chose a fixed time, every morning at 6:30, before breakfast, to take blood samples.

*^#^On May 30, we checked his CLZ blood concentration 5 times on the same day, and the results were as followed: 377.0 ng/ml (before taking medicine), 373.0 ng/ml (0.5 h after taking CLZ), 1,189.4 ng/ml (1 h after taking CLZ), 1,433.2 ng/ml (2 h after taking CLZ), 700.7 ng/ml (the following day at 6:30 AM, 10.5 h after taking the drug).*

**Figure 2:**
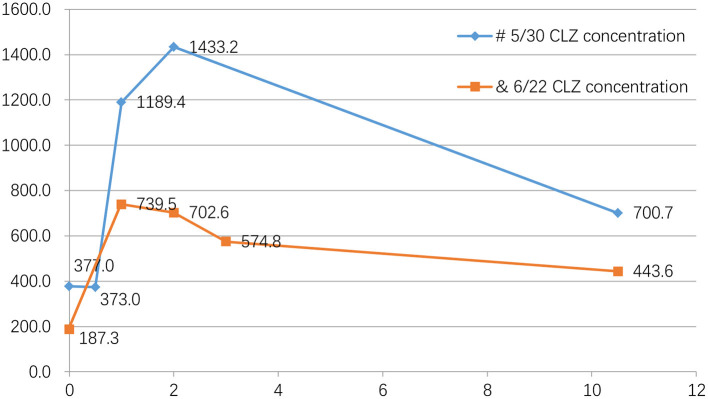
Blood concentration of clozapine five times on the same day. #On May 30, we checked his CLZ blood concentration 5 times on the same day, and the results were as followed: 377.0 ng/ml (before taking medicine), 373.0 ng/ml (0.5 h after taking CLZ), 1189.4 ng/ml (1 h after taking CLZ), 1433.2 ng/ml (2 h after taking CLZ), 700.7 ng/ml (the following day at 6:30 AM, 10.5 h after taking the drug). &On June 22, the results were as followed: 187.3 ng/ml (before taking medicine), 739.5 ng/ml (1 h after taking CLZ), 702.6 ng/ml (2 h after taking CLZ), 574.8 ng/ml (3 h after taking CLZ), 444.6 ng/ml (the following day at 6:30 AM, 10.5 h after taking CLZ) (Table 1; Figure 2).

*^&^On June 22, the results were as followed: 187.3 ng/ml (before taking medicine), 739.5 ng/ml (1 h after taking CLZ), 702.6 ng/ml (2 h after taking CLZ), 574.8 ng/ml (3 h after taking CLZ), 444.6 ng/ml (the following day at 6:30 AM, 10.5 h after taking CLZ) (Table 1 and Figure 2).*

*^a^At the time of the initial evaluation, when the patient was admitted at 13:00, 17 h after the last medication.*

*^b^For some reasons, the patient did not draw blood at 6:30 in the morning, but at 10:30, so this value is discrepant from others*.

## Diagnosis

According to the diagnostic criteria of the 10th edition of the International Classification of Diseases (ICD-10), the diagnosis is harmful use of clozapine, clonazepam dependence and schizophrenia.

## Therapeutic Intervention

The patient was admitted to the hospital on May 26, 2021. Considering his long-term use of CLZ and CLNAZ in large quantities, the patient was given glucose solution and other fluid support therapies on the first day of admission to promote the metabolism of CLZ, and continuously closely observed any changes in his mental state. At the same time, his drug dose was reduced to 34 tablets of CLZ (25 mg/tablet) and 10 tablets of CLNAZ (2 mg/tablet) per day, when the concentration of CLZ was 1,003.0 ng/ml. After that, 3 CLZ tablets and 2 CLNAZ tablets were reduced every 2 to 3 days, and we monitored the CLZ concentration approximately twice a week. We chose a fixed time, every morning at 6:30, before breakfast, to take blood samples (Table 1; Figure 1). On May 30, the third day of admission, he took 31 tablets of CLZ and 9 tablets of CLNAZ per day. Then, we checked his CLZ blood concentration 5 times on the same day, and the results were as followed: 377.0 ng/ml (before taking medicine), 373.0 ng/ml (0.5 h after taking CLZ), 1,189.4 ng/ml (1 h after taking CLZ), 1,433.2 ng/ml (2 h after taking CLZ), 700.7 ng/ml (the following day at 6:30 AM, 10.5 h after taking the drug) (Table 1; Figure 2). On June 22, the 28th day of admission, his CLZ dose was 18 tablets per day. We tested his CLZ concentration 5 times, and the results were as followed: 187.3 ng/ml (before taking medicine), 739.5 ng/ml (1 h after taking CLZ), 702.6 ng/ml (2 h after taking CLZ), 574.8 ng/ml (3 h after taking CLZ), 444.6 ng/ml (the following day at 6:30 AM, 10.5 h after taking CLZ) (Table 1; Figure 2). On June 12, 2021, when his CLZ was reduced to 4–12 tablets, the patient showed obvious symptoms of suspicion and anxiety. Therefore, we added the dose of CLZ back to 4–14 tablets on June 15. Considering the patient's ultra-rapid metabolism of CLZ, we adjusted the patient's CLZ dosing schedule from the original 4–14 tablets to 6–12 tablets. During the drug reduction process, the patient showed anxiety and restless sleep, so we gave him 50 mg trazodone every night, and then the anxiety was relieved. After one month of treatment, he was discharged from the hospital on July 1, 2021, and took CLZ 450 mg (6–12 tablets), CLNAZ 2 mg qn, and trazodone 50 mg qn.

## Outcomes and Follow-Up

In the course of drug reduction, the vital signs remained stable and the patient only experienced controllable anxiety and insomnia, with no other obvious withdrawal reaction. After being discharged from the hospital, the patient returned to the local hospital for further follow-up. Five months later, this patient accepted our follow-up by phone call. The patient is currently taking 18 CLZ tablets (450 mg) per day, the same amount when he was discharged, but he took CLNAZ 3 tablets (6 mg) per night and still showed mild anxiety and insomnia. He remained stable for about 5 months without recurrence of psychotic symptoms.

## Discussion

CLZ is an atypical antipsychotic drug that can reduce the mortality, hospitalization, and suicide risk of patients with refractory schizophrenia (10). In addition, it is the only effective drug for refractory schizophrenia. However, although CLZ is effective in treating psychosis, it may need to be discontinued in several cases such as pericarditis (11), agranulocytosis, and antipsychotic malignant syndrome (12). In addition, there may be a variety of withdrawal reactions such as autonomic nervous system symptoms, psychosis, gastrointestinal tract and serotonin discontinuation symptoms during drug withdrawal (5). So far, surprisingly, there are limited clinical guidance on how to minimize the likelihood of withdrawal symptoms in patients who stop CLZ treatment suddenly or gradually. Therefore, this case report can inform future clinical practice of adjusting CLZ treatment. According to the Arbeitsgemeinschaft für Neuropsychopharmakologie and Pharmakopsychiatrie (AGNP) neuropsychopharmacology TDM consensus guidelines (13), we gradually reduced CLZ by monitoring its serum concentration. For patients with overdose or suspected drug poisoning, detecting CLZ concentration once a day is not enough, at the beginning and end of drug withdrawal, we monitored his CLZ concentration 5 times on the same day to understand the changes in drug concentration before and after administration. By monitoring the drug concentration range, we successfully reduced the dose of CLZ, and rationally adjusted the daily dosing regimen according to the metabolic characteristics of CLZ. Our case provides a reference for patients with CLZ overdose to use TDM during drug withdrawal.

For this situation, the first thing we proposed is why the patient can use such a large dose of CLZ, without other noticeable side effects but hand tremors. One reason could be that he used rifampicin. Rifampicin was launched in the UK in 1967 and is licensed to treat infectious diseases, including tuberculosis. Rifampicin is an effective inducer of cytochrome P450, including isoenzymes CYP1A2 and 3A4. It is generally believed that it interacts with many drugs through these pathways, and its pharmacokinetics and interactions have been extensively reviewed (14, 15). Previous studies have shown an interaction between CLZ and rifampicin (16, 17), and the enzyme induction effect of rifampicin is very effective. It is necessary to increase the dose of CLZ by about 6 times to maintain the original treatment Effect. In addition, three examples support the above theory (18–20). Therefore, it is not difficult to explain why the patient received such a high dose of CLZ without noticeable side effects. When treating comorbidities in patients with CLZ, it is important to pay attention to drug interactions.

The second explanation is that the patient has an ultra-rapid metabolism genotype-CYP1A2. CLZ is metabolized in the liver by the cytochrome P450 (CYP450) enzyme superfamily (such as CYP1A2, CYP2D6, and CYP2C19). Among them, CYP1A2 enzyme is the main CYP enzyme involved in the metabolism of CLZ, and its activity is a potential determinant of CLZ dosage requirements (21, 22). We found that the patient carries the CYP1 Ultra-rapid metabolic genotype through drug gene testing, which indicates that the patient's CLZ metabolism is rapid. Therefore, it is necessary to provide a hefty dose of CLZ to maintain a better therapeutic effect. Pharmacogenetic testing can be combined with TDM to optimize treatment in certain specific cases.

It should be noted that the patient also over-dose CLNAZ while overdosing CLZ. Benzodiazepines are the most commonly prescribed psychotropic medications worldwide. The problem of use of benzodiazepines is related to abuse, dependence, and withdrawal syndrome. For patients with schizophrenia, CLZ is sometimes combined with CLNAZ, and the combination of the two can also help prevent the occurrence of tardive dyskinesia (TD) (23). In addition, some studies have also shown that increasing the use of CLNAZ can help improve the efficacy of CLZ and relieve anxiety symptoms in patients with schizophrenia (24). However, in the study conducted by Jiang P, they found that the combination of CLNAZ and CLZ can cause changes in blood CLZ and N-CLZ concentrations, and patients must increase the dose of CLZ to achieve satisfactory results (7). However, relevant research conclusions are not consistent. As there is no relevant guideline to reduce CLNAZ, we rely more on our clinical experience and the patients' clinical manifestations throughout the treatment process. We closely monitored the Clinical Institute Withdrawal Assessment scale-Benzodiazepines (CIWA-B) of patient and gradually reduced CLNAZ following the principle of “fast first, slow later.” Benzodiazepines abuse/dependence is a widespread and current problem, so more care should be taken when prescribing Benzodiazepines for patients with chronic insomnia.

Recently, scientific literature has shown the potential significance of Clozapine misuse/abuse, as well as the possible development of withdrawal syndrome. A study (3) on the 2005–2018 European Medicines Agency (EMA) Adverse Drug Reaction (ADR) data set showed that out of 11,847 CLZ-related ADRs, ~599 (5.05%) were related to misuse/ abuse/dependence/withdrawal issues, including 258 withdrawal-related (43.1%); 241 abuse-related (40.2%); and 80 ADRs related to deliberate abuse (13.3%). CLZ and its primary metabolite, N-CLZ, have on average the following pharmacological effects: (i) δ-opioid receptor agonist; (ii) Cannabinoid CB1 receptor agonist; (iii) Muscarinic receptor Body antagonist. These pharmacological activities are usually associated with the occurrence of pleasant effects, which may indicate the theoretical potential of CLZ to be abused by vulnerable individuals (3). In our case, the patient wanted to obtain the central nervous system clinical effects of the drug, such as drowsiness and sedation, so he kept adding the dose by himself. However, the possibility of abuse/dependence of CLZ needs further research. The high-dose intake, multidrug abuse of CLZ and CLNAZ reported here may be worth the attention of clinicians. Through this case, clinicians must pay attention to the abuse of prescription drugs such as CLZ and CLNAZ during the treatment of schizophrenia and insomnia.

Limitations: The data we show here is limited by clinical practice. The hospital cannot detect N-CLZ and CLNAZ. Otherwise, we will get more useful information. In addition, the interpretation of TDM results becomes more complicated when a dosing regimen that distributes daily doses in unequal ways is used. For example, the dose at night is higher than the dose during the day to achieve nighttime sedation. Third, the results of a single case may not apply to general patients, so the generalization of the results must be treated with caution.

## Conclusion

In summary, this case suggests (1) The blood concentration of CLZ is affected by genotype (such as CYP1A2), combination medication (such as rifampicin, CLNAZ), and other many other factors; (2) The reduction process of CLNAZ should follow the principle of “fast first and slow later,” and clinicans should closely monitor the patient's vital signs and drug withdrawal response; (3) Pharmacogenetic testing and TDM can help adjust the dosing regimen more rationally.

## Data Availability Statement

The original contributions presented in the study are included in the article/supplementary files, further inquiries can be directed to the corresponding author/s.

## Ethics Statement

Ethical review and approval was not required for the study on human participants in accordance with the local legislation and institutional requirements. The patients/participants provided their written informed consent to participate in this study. Written informed consent was obtained from the individual(s) for the publication of any potentially identifiable images or data included in this article.

## Author Contributions

WL, YL, and HW orchestrated the treatment and wrote the first draft of paper. HJ, JD, YZ, ZD, and SL edited the paper. All authors contributed to the article and approved the submitted version.

## Funding

This work was supported by Shanghai Mental Health Center Clinical Research Center Project (CRC2017ZD02), Shanghai Jiao Tong University Multidisciplinary Interdisciplinary Training Project (YG2019QNA10), Shanghai Jiao Tong University School of Medicine Curriculum Reform, Shanghai Flying Plan Funded Mental Health Center (2020-FX-03), and Shanghai Mental Health Center Project (2019-YJ11).

## Conflict of Interest

The authors declare that the research was conducted in the absence of any commercial or financial relationships that could be construed as a potential conflict of interest.

## Publisher's Note

All claims expressed in this article are solely those of the authors and do not necessarily represent those of their affiliated organizations, or those of the publisher, the editors and the reviewers. Any product that may be evaluated in this article, or claim that may be made by its manufacturer, is not guaranteed or endorsed by the publisher.
